# Patellar Fracture Repair Using FiberWire

**DOI:** 10.7759/cureus.44282

**Published:** 2023-08-28

**Authors:** Dhrupad Ponnamaneni, Rohan Mangal, Thor S Stead, Dwayne D'Souza, Latha Ganti

**Affiliations:** 1 Biomedical Sciences, University of Central Florida, Orlando, USA; 2 Medicine, University of Miami Miller School of Medicine, Miami, USA; 3 Medicine, The Warren Alpert Medical School of Brown University, Providence, USA; 4 Emergency Medicine and Orthopedics, Vanderbilt University Medical Center, Nashville, USA; 5 Medical Sciences, The Warren Alpert Medical School of Brown University, Providence, USA; 6 Emergency Medicine, University of Central Florida College of Medicine, Orlando, USA

**Keywords:** comminuted fracture, fiberwire®, open reduction and internal fixation, patella fracture repair, patella fracture

## Abstract

Patellar fractures are injuries caused by the direct impact on the bone or excessive stress on the extensor mechanism. The extensor mechanism is a structure formed by the quadriceps, the patella, and the patellar tendon, as well as ligaments. We present the case of a 53-year-old male who suffered a fall from a ladder after attempting to fix a ceiling light in his home. He went to the emergency department a few times before he was admitted due to his inability to walk secondary to a fracture located at the inferior pole of the left patella. This fracture was fixed with open reduction and internal fixation using drill holes and FiberWire®.

## Introduction

The patella, situated within the knee joint, is the largest sesamoid bone in the human body. The patella is encased in a thick cartilage layer [1]. The patella serves three essential functions: protecting the front of the knee, providing an attachment point for the quadriceps tendon, and acting as a fulcrum to optimize the efficiency of the extensor mechanism. Patellar fractures account for 1% of all fractures [2]. Direct contact on the bone or excessive strain on the extensor mechanism are two typical causes of patellar fractures. Patellar fractures can lead to extension weakness, stiffness, and patellofemoral arthritis [3]. The following classifications of patellar fractures are the most common that occur due to traumatic fractures of the patella: transverse, vertical, comminuted, marginal, and osteochondral [4]. They can also be comminuted, as was the case with our patient [5].

The treatment of patellar fractures can include the use of metal plates, tension-band wiring with metal such as Kirschner wire, or repair via drill holes and FiberWire® (Arthrex, Naples, FL, USA) [6]. In some cases, patellar fractures can be healed through non-surgical means if the fracture is non-displaced and has an intact extensor mechanism. Typically, this occurs using knee immobilization for a period of 8-10 weeks with serial radiographs to check on fracture healing. However, surgical fixation is indicated for patients with fractures that displace the extensor mechanism.

## Case presentation

**Figure 1:**
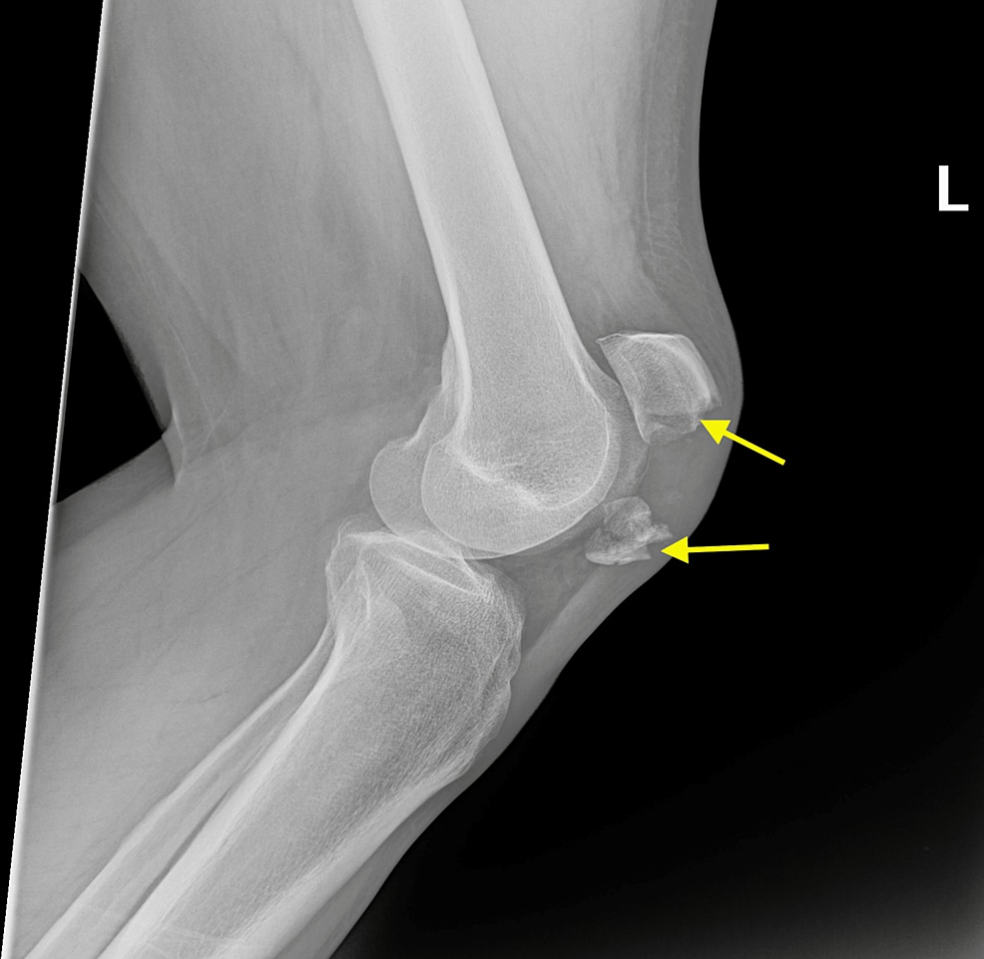
Radiograph demonstrating comminuted patella fracture

A 53-year-old male patient with no known medical condition or medical history presented to the emergency department (ED) for the third time with the same complaint of left knee pain. The patient fell from the ladder while fixing a ceiling light in his house the week prior to presentation. He slipped on the ladder and injured his left knee. He denied head injury, loss of consciousness, or any other injury. Left knee radiograph from the first visit showed an acute comminuted fracture through the inferior pole of the patella with superior distraction of the proximal fragment (Figure 1).

**Figure 2:**
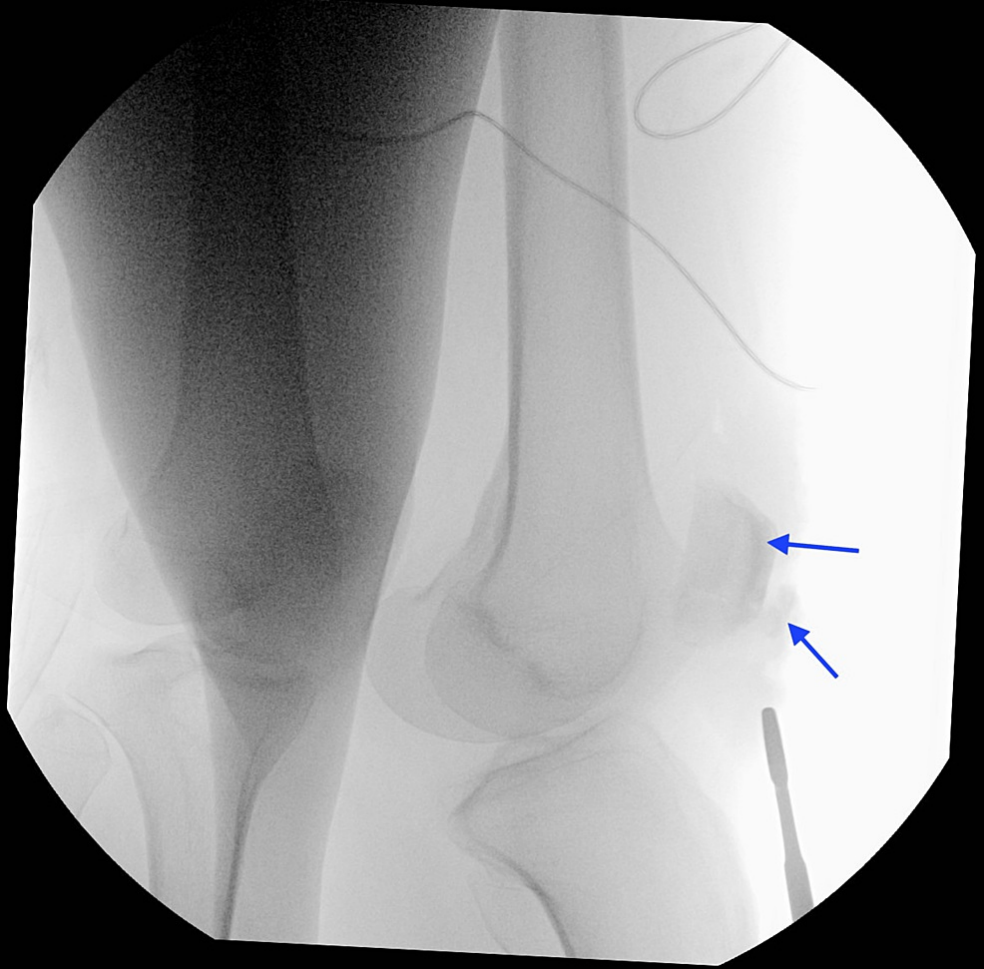
Intraoperative view of the patella under fluoroscopy

He was prescribed hydrocodone/acetaminophen for pain and sent home with crutches and instructions to follow up with an orthopedic surgeon. He came back to the ED the next day due to left knee pain and was prescribed a muscle relaxer and discharged home again. The patient came back to the ED for a third time with his spouse due to intractable left knee pain and inability to walk, and he also had run out of pain medication. This time, he was admitted to the orthopedic service where an open reduction and internal fixation (ORIF) was performed via fluoroscopy (Figure 2).

As the fracture site would not hold hardware, it was repaired using drill holes and FiberWire. FiberWire suture is constructed of a multi-strand, long-chain ultra-high molecular weight polyethylene (UHMWPE) core with a braided jacket of polyester and UHMWPE.

## Discussion

Patellar fractures can be repaired using hardware such as a metal plate, or via drill holes and specialized sutures as in this case. Some oppose the use of hardware, stating the high rate of postoperative complications [7]. One study argued that a modified Pyriford technique using a FiberWire suture would result in fewer postoperative complications than caused by a normal surgical process of using drill holes and FiberWire (similar to what procedure was performed in this case report) [7]. Furthermore, their research indicated that the modified tension band using FiberWire suture resulted in satisfactory clinical experience as well as fewer postoperative complications than an ORIF procedure. They finally concluded that FiberWire tension bands could be used in lieu of contemporary metal-wire tension bands to repair the patellar fracture. Another report highlighted the case of a 57-year-old patient who fell at work, causing a patellar fracture [6]. This fracture was treated with the use of a minimally invasive metal-free surgery rather than a traditional ORIF procedure with plates and screws. This report found that the patient was able to walk on his own 10 days after the surgery. Furthermore, this case report included a follow-up appointment that showed that the patient had no postoperative complications and that there was no need for suture removal.

## Conclusions

Patellar fractures are often first seen in the ED and typically require operative repair if displaced or the extensor mechanism is disrupted. The choice of operative technique can vary. In this case, it was demonstrated that using a strong suture with a high tensile strength versus typical repair using metal hardware resulted in a successful outcome.

## References

[1] Cox CF, Sinkler MA, Hubbard JB (2023). Anatomy, bony pelvis and lower limb, knee patella. StatPearls [Internet].

[2] Luo TD, Marino DV, Pilson H (2023). Patella fractures. StatPearls [Internet].

[3] Melvin JS, Mehta S (2011). Patellar fractures in adults. J Am Acad Orthop Surg.

[4] Gwinner C, Märdian S, Schwabe P, Schaser KD, Krapohl BD, Jung TM (2016). Current concepts review: fractures of the patella. GMS Interdiscip Plast Reconstr Surg DGPW.

[5] Chen R, Cao H, Sun Z, Jiang L, Li X, Zhao L, Liu X (2022). The clinical outcome of the reduction of the patellar inferior pole fracture with wire cerclage through a generated bone hole, in combination with patellar concentrator: a retrospective comparative study. J Orthop Surg Res.

[6] Hada S, Ishijima M, Tomita Y (2021). A case report of patellar fracture treated by percutaneous strong suture technique. Ann Med Surg (Lond).

[7] Egol K, Howard D, Monroy A, Crespo A, Tejwani N, Davidovitch R (2014). Patella fracture fixation with suture and wire: you reap what you sew. Iowa Orthop J.

